# A life for autoimmune blistering diseases: in memoriam Detlef Zillikens

**DOI:** 10.3389/fimmu.2023.1291590

**Published:** 2023-10-19

**Authors:** Jennifer E. Hundt, Christian D. Sadik, Nina van Beek, Hauke Busch, Frédéric Caux, Matthias Goebeler, Christoph M. Hammers, Karin Hartmann, Takashi Hashimoto, Saleh Ibrahim, Michael Kasperkiewicz, Dedee F. Murrell, Andreas Recke, Christian Rose, Nina Schumacher, Iakov Shimanovich, Cassian Sitaru, Patrick Terheyden, Diamant Thaçi, Ralf J. Ludwig, Enno Schmidt

**Affiliations:** ^1^ Lübeck Institute of Experimental Dermatology (LIED), University of Lübeck, Lübeck, Germany; ^2^ Department of Dermatology, University of Lübeck, Lübeck, Germany; ^3^ Department of Dermatology, Groupe Hospitalier Paris Seine-Saint-Denis, AP-HP, Bobigny, France; ^4^ Department of Dermatology, University of Würzburg, Würzburg, Germany; ^5^ Department of Dermatology, University of Kiel, Kiel, Germany; ^6^ Department of Dermatology, Basel, Switzerland; ^7^ Department of Dermatology, Osaka Metropolitan University Graduate School of Medicine, Osaka, Japan; ^8^ Khalifa University, Abu Dhabi, United Arab Emirates; ^9^ Department of Dermatology, Keck School of Medicine, University of Southern California, Los Angeles, CA, United States; ^10^ Department of Dermatology, St George Hospital, University of New South Wales (UNSW), Sydney, NSW, Australia; ^11^ Dermatohistologisches Einsendelabor Lübeck, Lübeck, Germany; ^12^ Department of Dermatology, Faculty of Medicine, Medical Center-University of Freiburg, Freiburg, Germany; ^13^ Institute and Comprehensive Center for Inflammation Medicine (CCIM), University of Lübeck, Lübeck, Germany

**Keywords:** autoimmune blistering diseases, pemphigoid, pemphigus, autoantibody, immuno-fluorescence, dermatology, clinician scientists

## Abstract

Detlef Zillikens, MD, director and chair of the Department of Dermatology at the University of Lübeck, Lübeck, Germany, died in September 2022, aged only 64. He dedicated his professional life to autoimmune blistering diseases (AIBDs) and built his department into one of the world’s leading centers for these diseases. Herein, his professional life and the impact on the field of AIBDs and the research landscape at the University of Lübeck are addressed. With his warm, integrative, open-minded, ever-optimistic attitude, he was a highly reliable colleague, mentor, and friend to many in the field including each of the authors. Combined with his in-depth knowledge of dermatology, interest in many fields of life science, and hard work, Detlef Zillikens initiated the founding of two independent research institutes, the Lübeck Institute of Experimental Dermatology and the Institute and Comprehensive Center for Inflammation Medicine. He was also instrumental in establishing the Center for Research on Inflammation of the Skin, where in a new research building, over 140 scientists pursue research questions related to skin inflammation. By inviting numerous researchers and clinicians to his department and hosting two large international meetings, he brought the field of AIBDs much closer together and inspired multiple national and international research initiatives. His ideas will live on and grow in many of his colleagues and mentees.

## Introduction

**Figure 1:**
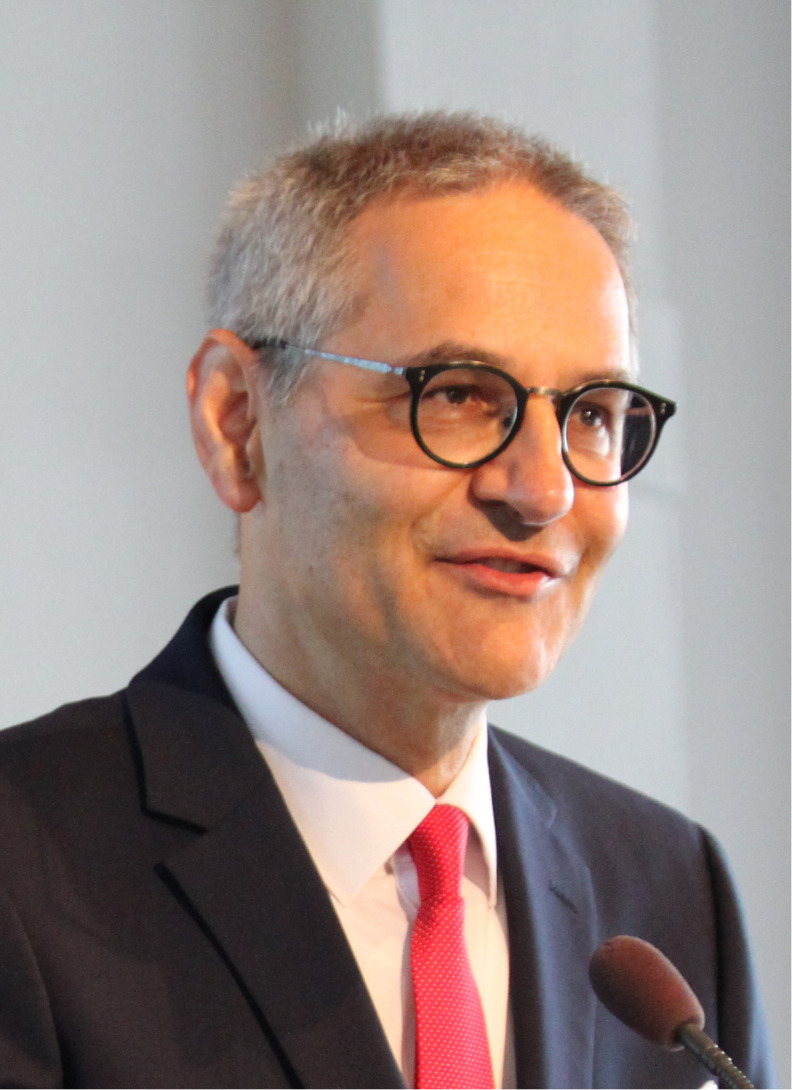
Detlef Zillikens, July 15, 1958, to September 19, 2022.

Detlef Zillikens, MD, died on September 19, 2022, aged 64, after being diagnosed with a glioblastoma in March of that year (**Figure 1**). Following emergency surgery, he could not return to the office. Detlef Zillikens was the director and chair of the Department of Dermatology, Allergology and Venereology at the University of Lübeck, Lübeck, Germany. He was a leading expert in autoimmune blistering diseases (AIBDs) and has established the world’s most prominent research center for these disorders in Lübeck. Detlef Zillikens was an optimistic, warm, integrative, and highly reliable colleague, mentor, teacher, and friend to us authors. He was a great motivator and an extraordinarily hard-working clinician and researcher who consistently followed his principles and goals. We will never forget his broad knowledge, passion for research, patience, enthusiasm, empathy, smiling face, and warm-hearted character. His death left all of us stunned and deeply saddened. We will now strive to continue and further develop the enormously successful path he has led us. Some part of Detlef Zillikens will always live on in our work and hearts. His life is honoured by the naming of the Clinician Scientist Academy of the Universities of Lübeck and Kiel after his person.

Several obituaries have already highlighted and valued his scientific work (1–4). Here, within the Research Topic *Autoimmune Blistering Diseases: in memoriam Detlef Zillikens*, numerous colleagues presented their research and how they were connected and inspired by Detlef Zillikens to focus on AIBDs. Below, we take a closer look at his life and work from different angles and perspectives.

## Curriculum vitae

Detlef Zillikens grew up in Wesseling near Bonn, Germany, with two sisters, his mother, who worked as a lab technician, and his father, who held a PhD in chemistry. After schooling in Wesseling and later in Bonn, he went to medical school at the Universities of Bonn and Heidelberg and, in his final year, to the University of Illinois at Champaign-Urbana, USA, where he gained his fine Midwestern American accent. His medical thesis (Dr. med.) was performed at the Institute of Clinical Chemistry, University of Heidelberg (Prof. H. Schmidt-Gayk), where he developed a radioimmunoassay to detect human parathyroid hormone. In 1986, he began a residency at the Department of General Surgery in Heppenheim, and he—fortunately—moved into Dermatology in 1988 to start his residency at the Department of Dermatology, University of Würzburg, Würzburg, Germany, under the guidance of Prof. Günther Burg. In 1992, the Department was chaired by Prof. Eva-Bettina Bröcker; just a week after passing his board examination, he became a consultant. In the same year, he published his first research papers about AIBDs (5, 6). After his board certification in allergology and phlebology, he habilitated in 1994 on “Cellular and humoral immune mechanisms in the pathogenesis of bullous pemphigoid and pemphigus”, becoming an assistant professor. With a prestigious grant from the German Research Foundation (*Deutsche Forschungsgemeinschaft* (DFG)), he joined the laboratory of Prof. Luis Diaz and Dr. George Giudice, Medical College of Wisconsin, Milwaukee, USA, from 1994 to 1997. During this time, Detlef Zillikens characterized the fine specificities of autoantibodies against BP180 (type XVII collagen) in different pemphigoid diseases. Back in Würzburg, he became an associate professor in 2000 and full professor in 2003.

In April 2004, Detlef Zillikens was appointed director and chair of the Dermatology Department at the University of Lübeck. In parallel, while energetically restructuring and considerably enlarging the clinical department, he implemented his major field of interest, the AIBDs, in the research agenda of the University of Lübeck. Since 2007, he acted as principal investigator and member of the steering committee of the Cluster of Excellence 303 *Inflammation at Interfaces*, followed since 2019 by the Cluster of Excellence 2167 *Precision Medicine in Chronic Inflammation*. The Clusters of Excellence are the largest funding instruments of the German Research Foundation. Through this initiative, five additional professorships were taken by R.J.L. (2008), S.I. (2009), and Manfred Kunz (2009), followed by D.T. (2013) and then H.B. (2016) and J.H. (2018). Subsequently, two independent institutes were established, the Institute and Comprehensive Center for Inflammation Medicine (CCIM; 2009–2010, director, Manfred Kunz; 2010–2013, acting director, E.S.; since 2013, director, D.T.) and, in 2014, the Lübeck Institute of Experimental Dermatology (LIED) with its four divisions: *Model Systems*, director, R.L. and J.H.; *Genetics*, director, S.I.; *Translation*, director, E.S. (endowed professorship 2014); and *Systems Biology*, director, H.B.

Detlef Zillikens initiated and led several large research consortia, initially, the Focus Program *Autoimmunity* by internal funding (2007–2011) being the nucleus of the DFG-funded research consortia comprising the Research Training Group 1727 *Modulation of Autoimmunity* (2011–2020), the Clinical Research Group 303 *Pemphigoid Diseases* (2016–2021), and finally, starting in 2022, the Collaborative Research Center 1526 *Pathomechanisms of Antibody-mediated Autoimmunity: Insights from Pemphigoid Diseases*. By the CRC 303, another professorship taken by C.S. was brought to his department in 2016.

With his kind and integrative manner, Detlef Zillikens attracted the interest in AIBDs of other institutes at the University of Lübeck, such as the Departments of Anatomy, Microbiology, Pharmacology, Nephrology, and Immunology. Currently, more than 20 clinicians and scientists work in the field of AIBDs on the Lübeck Campus outside the Department of Dermatology, CCIM, and LIED. To bring these people together under one roof, he secured funding for a 2,500-m^2^ research building in 2017, the *Center for Research on Inflammation of the Skin* (CRIS). Unfortunately, Detlef Zillikens could not enjoy the opening of this center scheduled for 2026.

Apart from basic science and clinical dermatology, two other areas were close to his heart, the development of diagnostic tools for AIBDs and the training of clinician scientists. The long-standing and very fruitful scientific cooperation with the company Euroimmun, Lübeck, resulted in the availability of various serological assays for AIBDs that have greatly facilitated the diagnosis of these disorders. For the training of clinician scientists, he acquired DFG funding that led to the foundation of the *Lübeck School for Clinician Scientists* and negotiated a 6-year curriculum with the board of physicians of Schleswig-Holstein combining a 4-year clinical training with a 2-year research rotation.

Detlef Zillikens served as an elected member of the review board for medicine of the DFG (2012–2020) and as vice president of the University of Lübeck responsible for scientific activities from 2013 to 2018. Detlef Zillikens received numerous prizes and honors and co-authored more than 600 articles and book chapters in the field of inflammatory dermatoses.

Detlef Zillikens leaves behind his wife Monika, a psychiatrist and psychotherapist, and two children, currently in dermatological resident training and medical school.

## Department of Dermatology, University of Lübeck

Under the care of 57 current physicians, the Department of Dermatology operates 62 beds on two wards and a day-care clinic that serves more than 10,000 patients per year. In 2004, when he became chair of the Department, only 17 physicians and 32 beds were present. A continuous innovative educational program based on a rotation principle through five different inpatient and outpatient teams imparts all the essential learning content of dermatology. The expansion of the research structures was accompanied by the establishment of special outpatient clinics for AIBDs (four clinics/week), skin cancer, phlebology, and chronic wounds and a section for inflammatory disease with a focus on atopic dermatitis, psoriasis, and urticaria. The clinic has a certified skin cancer center and is part of the Lübeck University Vascular and Cancer Centers. Other important pillars of clinical care are the division of allergology, the dermatopathology laboratory, and the routine autoimmune laboratory. Detlef Zillikens has particularly promoted and always supported dermatopathology, a section that constantly expanded during his guidance. Linking the autoimmune serology, direct immunofluorescence, and histopathology, the detailed histopathological features of anti-p200 and anti-laminin 332 pemphigoid were described (7, 8). The autoimmune laboratory in 2010 was the first dermatology laboratory in Germany to be ISO 15189 certified and developed into a national and international reference center for AIBDs (9). In 2022, the laboratory received AIBD samples from more than 400 sites in Germany and abroad and processed 7,000 serum and more than 2,000 tissue samples for direct immunofluorescence. Detlef Zillikens signed all reports personally and educated and supervised a team of motivated residents and junior consultants who read the immunofluorescence slides. He was in constant exchange with the dedicated technical team that valued him highly.

## Institute and Comprehensive Center for Inflammation Medicine

**Figure 2:**
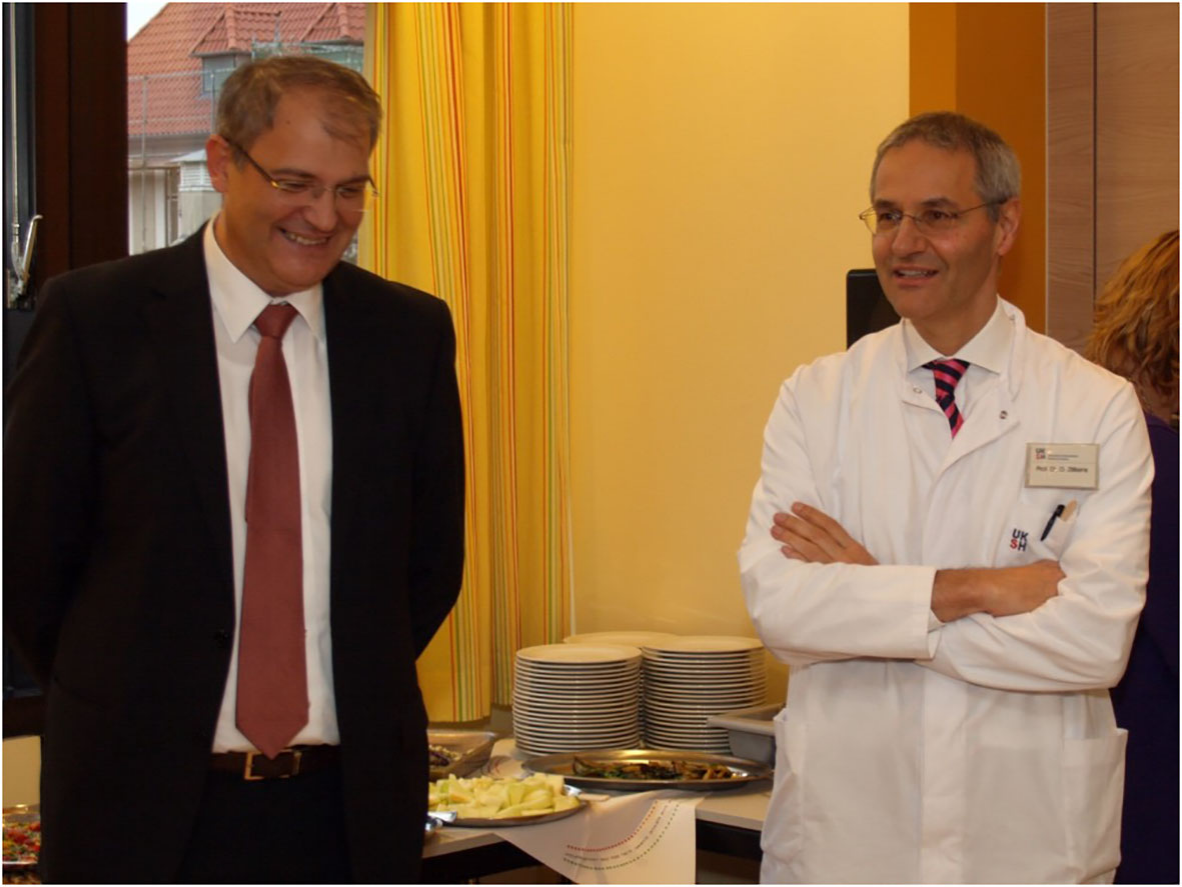
Inauguration of Diamant Thaci (left) to CCIM in 2013 (right, Detlef Zillikens). CCIM, Comprehensive Center for Inflammation Medicine.

During the first period of the Excellence Cluster *Inflammation at Interfaces*, the idea of establishing a center of excellence in Lübeck was supported very early by Detlef Zillikens. In the beginning, it was organized to have a coordinating function between basic research activities and interdisciplinary scientific projects. Following the appointment of D.T. as its director in 2013 (**Figure 2**) under the constant support of Detlef Zillikens, CCIM grew into an interdisciplinary center of excellence in the management of patients with common chronic inflammatory skin diseases, e.g., psoriasis, hidradenitis suppurativa, or atopic dermatitis, and also more complex cases requiring highly specialized approaches in diagnosing and treatment. In interdisciplinary joined clinics, e.g., together with rheumatologists, all patients were screened for the existence of associated musculoskeletal diseases. Later, other disciplines joined like gastroenterology, nutrition medicines, and infectious diseases. Another focus of CCIM is patient-oriented clinical research including randomized controlled trials (10–12). In 2018, Detlef Zillikens supported the founding of a research institute for inflammation medicine and, within the Department of Dermatology, a section for inflammatory skin diseases. All of more than 40 coworkers of the research institute and section of dermatology considered him a strong supporter, visionary, and pioneer of modern dermatology.

## Lübeck Institute of Experimental Dermatology

**Figure 3:**
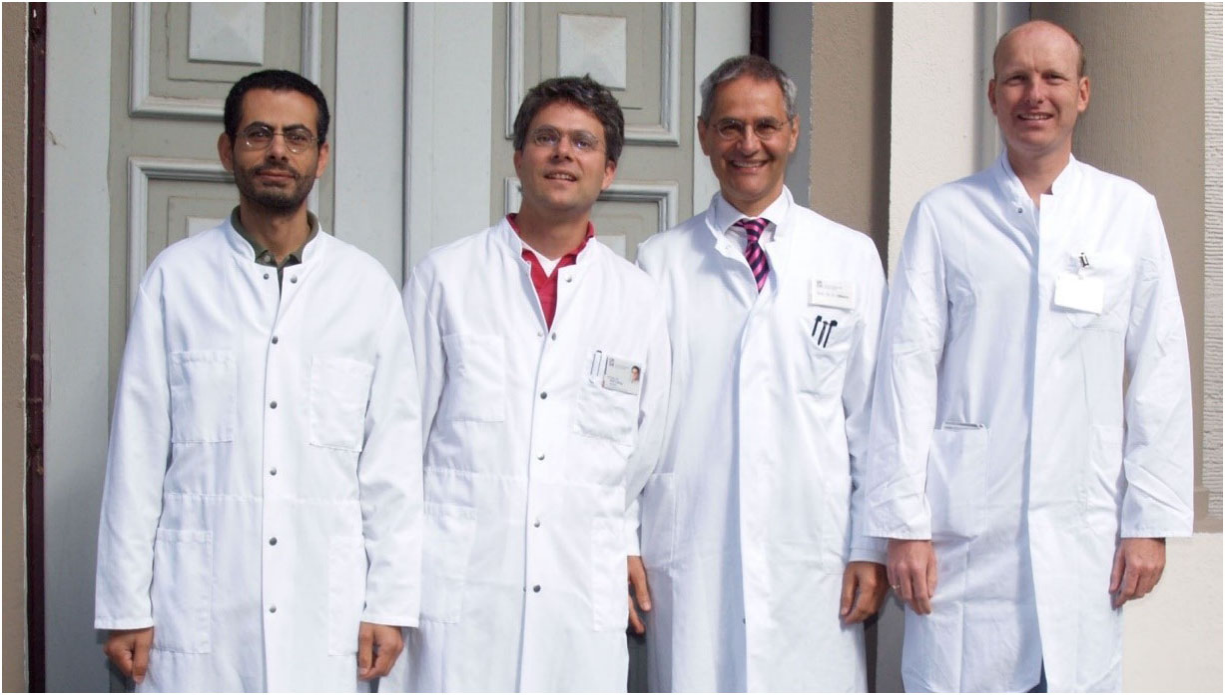
Founding professors and directors of the Lübeck Institute of Experimental Dermatology in 2014 (Saleh Ibrahim, Ralf Ludwig, Detlef Zillikens, and Enno Schmidt, from left to right).

Detlef Zillikens’ research agenda on AIBDs directly contributed to the scientific focus of the University of Lübeck, namely, infection and inflammation. With the acquisition of two professorships within the Excellence Cluster *Inflammation at Interfaces* and an endowed professorship, it became possible in 2014 to further boost research in this direction by founding this institute as a spin-off of the Department of Dermatology (**Figure 3**). Translational research on inflammation of the skin has been intensified ever since and extended to other inflammatory skin diseases such as psoriasis and atopic eczema. Together with two additional professorships established within the Excellence Cluster in 2016 and 2018, the LIED harbored five professorships, J.EH., H.B., S.I., R.J.L., and E.S., within its four divisions, *Model Systems* (R.J.L. and J.E.H.), *Genetics* (S.I.; until 2022), *Translation* (E.S.), and *Systems Biology* (H.B.).

Its research comprises a broad scope including various *in vitro*, *ex vivo*, and *in vivo* models, imaging, genetics, epidemiology, diagnostics of AIBDs, and systems biology. Research on LIED is centered around but not restricted to AIBDs.

LIED has established strong cooperation with the University of Lübeck and the Excellence Cluster *Inflammation at Interfaces* and its continuation *Precision Medicine in Chronic Inflammation* and has achieved national and international visibility. This development is reflected in the participation in various third-party funded consortia such as the Research Training Groups 1727 *Modulation of Autoimmunity*, 1743 *Genes, Environment, and Inflammation*, and 2633 *Defining and Targeting Autoimmune Pre-Disease*, as well as in the Clinical Research Group 303 *Pemphigoid Diseases* and its successor CRC 1526 *Pathomechanisms of Antibody-mediated Autoimmunity*. LIED also combines basic research with clinical research in close cooperation with the Department of Dermatology and the CCIM. The laboratories of LIED house more than 60 scientists, clinician scientists, PhD students, and technicians, being located at three sites on the campus. With the newly acquired CRIS building, the LIED members will be united under one roof in 2026.

## Research consortia

### Research Training Group 1727 Modulation of Autoimmunity

**Figure 4:**
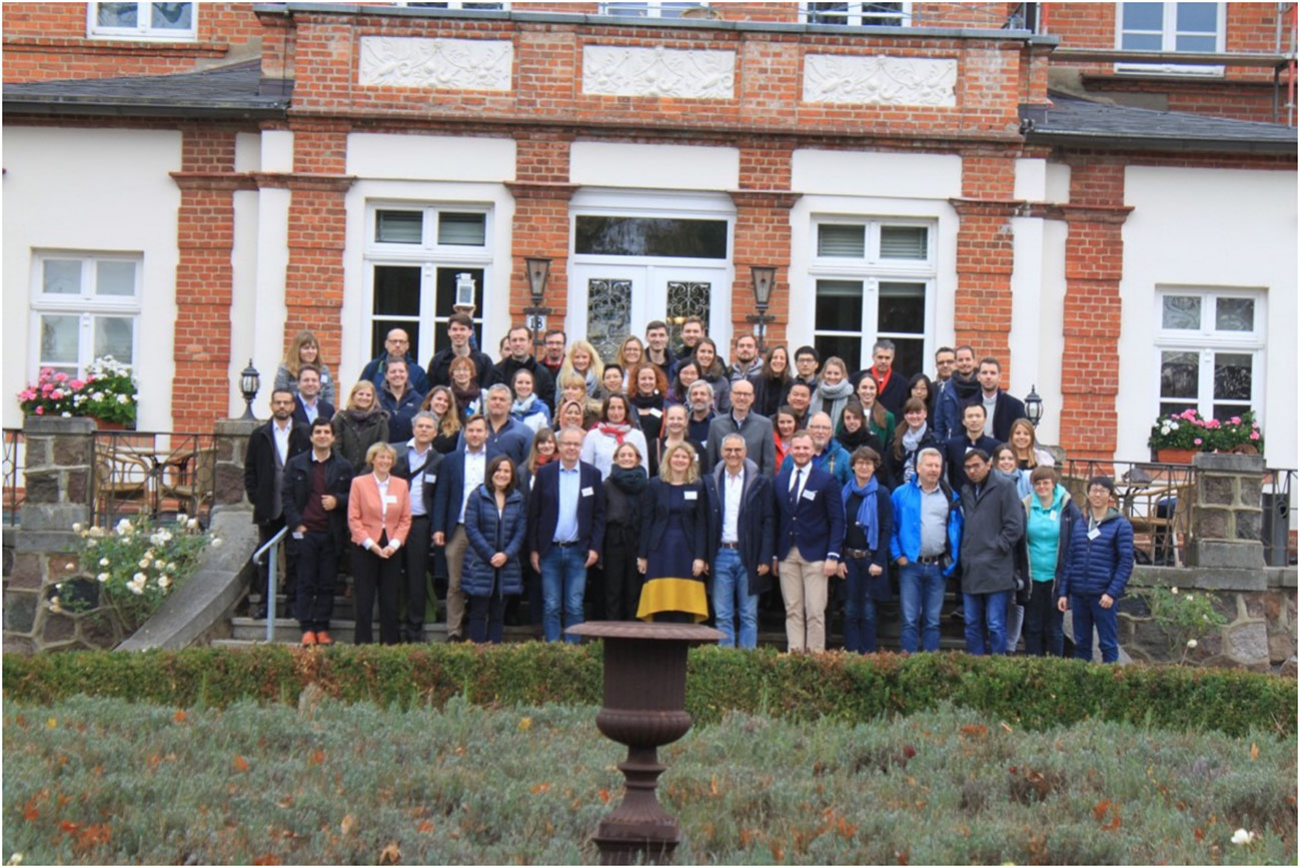
Meeting of the Research Training Group 1727 in Boltenhagen, Germany, in 2016.

The interdisciplinary projects for this Research Training Group (RTG) have evolved from the Priority Program on Autoimmunity, which was funded by the University of Lübeck from 2006 to 2011. The central scientific question of the RTG addressed the loss of immune tolerance in autoimmune diseases and the modulation of the mechanisms contributing to this loss. The projects were divided into two areas: A) the development of novel therapeutic strategies for the treatment of autoimmune diseases and B) the identification of novel therapeutic targets. The scientists involved in this RTG have been collaborating on this topic at the Lübeck campus for more than 10 years. The breadth of the spectrum of methods used in this work also served as a training program for doctoral students and is supplemented by a central qualification and supervision concept with defined training modules. This included a twice-yearly retreat where all students present their respective projects and receive feedback from the group of subproject leaders and external advisors (**Figure 4**). Principal investigators came from the Departments of Dermatology, LIED, Rheumatology, Immunology, Anatomy, Microbiology, and Chemistry. During the funding period of 2015–2020, three generations of 13 Dr. rer. nat./PhD students were trained. In addition, six to 12 scholarships per year were provided for medical students who pause their medical studies for 6–12 months to focus exclusively on an experimental MD thesis. In total, 36 PhD and 30 associated PhD students funded by other third-party funds as well as 80 MD and associated MD students received their degrees through this RTG.

The speaker of this RTG was Detlef Zillikens; scientific coordinators in the first funding phase were R.J.L. and in the second funding phase J.E.H. This funding instrument has also been particularly successful in getting young medical students interested in experimental research on autoimmunity. A number of PhD students have joined the Clinician Scientist Program of the clinic in their later training or continued their research as postdocs at the Lübeck campus.

### Clinical Research Group 303 Pemphigoid Diseases and Collaborative Research Center 1526 Pathomechanisms of Antibody-mediated Autoimmunity: Insights from Pemphigoid diseases

In 2012, Detlef Zillikens recruited C.D.S. from the Center of Immunology and Inflammatory Diseases of the Massachusetts General Hospital/Harvard Medical School in Boston to Lübeck. He had been working at this point on the mechanisms regulating the recruitment of granulocytes in autoimmune diseases for 4 years. The mechanisms recruiting granulocytes in pemphigoid diseases thus became more of the focus of the research community at Detlef Zillikens’ Department. With Detlef Zillikens’ support, C.D.S. drove the development of a DFG-funded research consortium focused on the mechanism regulating granulocyte recruitment in the early stages of the effector phase of pemphigoid diseases as paradigm diseases for antibody-induced granulocytic tissue inflammation. This research consortium, the Clinical Research Unit (CRU) 303 *Pemphigoid Diseases—Molecular Pathways and their therapeutic Potential*, ran from 2015 to 2022. It consisted of nine research projects and two service projects with researchers from multiple departments of the University of Lübeck as well as from the Max Planck Institute for Evolutionary Biology, Plön. Through the translational research program of the CRU 303, the clinic and the research of the Department of Dermatology in Lübeck became more closely intertwined with the CRU, providing the resources to profile and sample patients with pemphigoid diseases at clinical and molecular levels in a highly standardized manner and giving many clinical fellows of the department the chance to engage into research rotation in the labs participating in the CRU 303.

The CRC 1526 succeeded the CRU 303. CRCs are in general larger funding instruments of the DFG than CRUs. They are meant to run for 12 years and allow more projects and research sites to engage in a concerted research effort than CRUs do. The CRC 1526 addresses pemphigoid diseases and other autoantibody-induced diseases on a broader scope. The CRC 1526 became operative in January 2022 just 2 months before Detlef Zillikens was diagnosed as terminally ill.

### Center for Research on Inflammation of the Skin

The CRIS is an interdisciplinary research center with a thematic focus on chronic inflammatory processes of the skin. The research program of CRIS includes the elucidation of the molecular mechanisms underlying these processes, the uncovering of differences in the mechanisms of the various types of chronic inflammation of the skin, and the development of novel, selective strategies to interrupt and reverse these mechanisms for therapeutic purposes. The CRIS research building is currently under construction on the campus of the University of Lübeck. Its completion and occupancy are planned for 2026. The building will provide 2,500 m^2^ of space for 140 scientists to implement the CRIS research program. In addition to state-of-the-art laboratory space, the building will house a research clinic and animal facility and several high-end equipment including a CyTOF mass cytometer and a mass spectrometry imaging system.

A total of €30 million will be made available for the construction and furnishing of the CRIS research building. The funding commitment in 2017 was made from federal and state funds on the basis of a competitive, two-stage application procedure coordinated by the Science Council in accordance with Article 91b of the German Basic Law. This application procedure promotes innovative research consortia that are to develop into international beacons. The 10-member interdisciplinary applicant group of scientists from the Lübeck campus was led by Detlef Zillikens and managed by C.D.S.

### Clinician scientist program

The training of clinician scientists was always paramount for Detlef Zillikens. As such, within the last 15 years, more than 25 clinician scientists including N.v.B, C.M.M, M.K., A.R., and I.S. received their dermatological education and were enabled to perform research projects on the Lübeck campus. During his time as vice president of the University of Lübeck, Detlef Zillikens initiated a competitive funding program for dedicated physicians of all clinical disciplines, which allowed several of these clinical scientists to perform their research rotations. Detlef Zillikens had negotiated the dermatological training program with the Schleswig-Holstein Medical Association. The 5-year, originally purely clinical dermatological training curriculum was extended to 6 years but integrates 2 years of protected time for research. Due to his longstanding and exceptional input in this subject and as a lasting memory, the *Clinician Scientist Academy* of the Universities of Lübeck and Kiel was named after him.

## Cooperation with Euroimmun

Soon after Detlef Zillikens started his position in Lübeck, he sought contact with Euroimmun, an aspiring and rapidly expanding company for autoimmune diagnostics situated in Lübeck. In close cooperation with Winfried Stöcker, former owner and CEO of Euroimmun, Wolfgang Schlumberger, the former CEO, and highly motivated and forward-thinking group leaders, e.g., Christian Probst, Lars Komorowski, and Cornelia Dähnrich, a variety of serological assays for the diagnosis of AIBDs were developed and became widely available. As such, employing the recombinant immunodominant fragments sensitive and specific ELISA for the detection of serum autoantibodies against major AIBD target antigens were released comprising BP180 (type VII collagen), BP230, desmoglein 1 and 3, envoplakin, type VII collagen, tissue-type transglutaminase, and deamidated gliadin (13–18). Based on the Biochip^®^ technology, in which multiple miniature substrates are placed in a single incubation field mounted to a standard laboratory slide, additional assays were developed, e.g., the Biochip^®^ mosaic containing monkey esophagus, primate salt-split skin, recombinant BP180 NC16A, and human HEK293 cells that recombinantly express the immunodominant regions of desmoglein 1, desmoglein 3, and BP230 on their surface (19). This Biochip^®^ mosaic is now widely used and has further been extended by cells that express type VII collagen and laminin 332 (20–28). Future studies will address the detection of serum IgG against further target antigens such as the p200 protein, the ectodomain of BP180, and IgA autoantibodies in AIBDs. The cooperation with Euroimmun reflected Detlef Zillikens’s ability to interest others in his agenda, to build bridges, to create win–win situations, and to convey a constructive and warm atmosphere on an equal footing. This cooperation was always among his highest priorities and emphasized his strong translational thinking.

## Research

Already during residency, Detlef Zillikens became interested in AIBDs. When he was appointed head of the routine autoimmune laboratory at the Department of Dermatology in Würzburg in 1992, immediately after receiving his board certification in dermatology, he started his own research group. Without funding and experimental background, apart from his activities as a medical student, this was built on medical students and situated in the three rooms of the routine autoimmune laboratory. Here, Andreas Ambach, Achim Zentner, and E.S. were among his first students who performed their medical theses (to receive the title M.D. that in Germany is not automatically given after the final medical exam). At that time, he was mainly interested in inflammatory mediators in the sera and blister fluids of patients with the most frequent AIBDs, bullous pemphigoid. Meanwhile, Detlef Zillikens was working full time as a clinical consultant spending his evening and weekend hours with research activities. The latter was only possible through the dedicated team of the routine autoimmune laboratory that also greatly supported the medical students and highly valued Detlef Zillikens. The data of these medical students were meticulously published and, given the limited resources at that time, reached considerable attention (5, 6, 29–32).

Supported by a prestigious research grant from the DFG, he spent 3 years (1994–1997) in the laboratory of Prof. Luis Diaz, Milwaukee, WI, USA. He initially aimed to study the neonatal mouse model of bullous pemphigoid, established in this lab in 1993 (33). To his great disappointment, this did not work out, and together with Dr. George Giudice, he instead characterized the fine specificities of autoantibodies against BP180 (type XVII collagen) in different pemphigoid diseases by the use of various recombinant fragments of the immunodominant NC16A domain (34–37).

Back in Würzburg, he re-established his research group that initially included the part-time technician Heike Schömig and E.S., who decided to start residency in dermatology with a 1-year research rotation as, what today would be called a clinician scientist. At this time, anti-BP180 IgG was shown to trigger a signal-transducing event leading to the release of IL-6 and IL-8 when incubated with cultured human keratinocytes (detailed below) (38). By Detlef Zillikens’ winning, caring, and binding character, the laboratory was seen populated with a number of highly motivated and dedicated medical students, including I.S., Karin Obe, Karin Herzele, Rebeccah Döpp, Stanislav Reimer, Werner Kippes, Robert Kränsel, and Matthias Georgi (37, 39–49). These activities were soon supported by the implementation of a so-called “tandem” system. Here, Detlef Zillikens and M.G., another talented and aspiring consultant at that time and current director and chair of the Department of Dermatology, University of Würzburg, Germany, covered a single clinical position, allowing them to exclusively dedicate about 4 months per year to research. With this system, Prof. Eva-Bettina Bröcker fueled their careers and anticipated the *advanced clinician scientist* by nearly 20 years. When E.S. moved to the Department of Biotechnology for his PhD in 1999, C.S. took over as manager of Detlef Zillikens’ research laboratory.

Detlef Zillikens was involved as a supervisor in the DFG-funded Graduate Program *Immunomodulation*, a training program for students of the Graduate School of Life Sciences (GSLS) and medical students of the University of Würzburg. C.S. and later Sidonia Mihai were recruited to Detlef’s lab as fellows in this program. C.S. together with Katrin Müller-Blech as a technical assistant, Yoshi Hirako as a postdoc, and several active medical students worked on an *ex vivo* model of autoantibody-induced dermal–epidermal separation. This model was used in subsequent studies to address the effector phase of the autoimmune response to components of the epidermal basement membrane (50, 51). In parallel, animal models of autoimmunity to collagen VII were developed (detailed below) (52, 53).

In Lübeck, the research group considerably expanded with the postdocs and clinician scientists including I.S., Mircea Chiriac, Andreas Recke, Josep Herrero-González, Alina Sesarman, Amrei Dilling, and Bernie Gibbs. The results obtained in these projects were the basis for participation in the DFG Cluster of Excellence *Inflammation at Interfaces*, which started in 2007.

After R.J.L. and E.S. joined the Department of Dermatology in Lübeck in October 2007 and January 2008, respectively, Detlef Zillikens retracted from everyday research activities and focused on serving in various functions to foster his research ideas as detailed below. In addition, he initiated and steered the above-mentioned research consortia that enabled more than 70 third-party-funded clinician scientists and researchers at the campus Lübeck to work on AIBDs in 2022. Nevertheless, he attended the weekly major laboratory meetings, gave highly appreciated input in nearly every project, and reviewed the resulting manuscripts. At the same time, he fostered the work of clinician scientists represented here by M.K., A.R., and N.v.B. as further detailed in the obituary of C.M.H. (3). Detlef Zillikens personally selected them from a large number of applicants and closely mentored their careers.

Below, the authors have detailed those articles from his more than 600 publications that have been highly cited and/or are regarded as most relevant for the current research activities both in Lübeck and internationally. Among his many review articles, the three most cited were *Pemphigoid diseases* in *Lancet* 2013 (54), *Pemphigus* in *Nature Reviews Disease Primers* in 2017 (55), and *Modern diagnosis of autoimmune blistering diseases* in *Autoimmunity Reviews* in 2010 (56). He also co-authored highly cited consensus and management guidelines of AIBDs (57–68) and was instrumental in generating the guidelines for the use of immunoadsorption and rituximab. Consensus recommendations for the latter therapies, at this time, highly innovative therapeutic options for severe and/or refractory AIBDs, have greatly facilitated the reimbursement of the treatment costs in Germany (69, 70).

## Selected publications

### A novel subepidermal blistering disease with autoantibodies to a 200-kDa antigen of the basement membrane zone, and Anti-laminin γ1 pemphigoid

In 1996, Detlef Zillikens and T.H. described the first patient with anti-p200 pemphigoid (71). The patients clinically resembled bullous pemphigoid and also showed linear deposits of IgG and C3 along the dermal–epidermal junction. In contrast to bullous pemphigoid, serum IgG bound to the floor of the artificial blister by indirect IF microscopy on human salt-split skin as previously described for epidermolysis bullosa acquisita. In contrast to the latter disorder, the patient’s serum reacted with a specific 200-kDa band by immunoblotting with dermal extract. The molecular identity of the target antigen was only revealed in 2009 by the group of T.H. (72), who described laminin γ1 to be recognized by 90% of anti-p200 pemphigoid sera. Subsequently, it became clear that only 70%–90% of anti-p200 pemphigoid sera are reactive with laminin γ1 and that the pathogenicity of anti-laminin γ1 IgG *in vivo* could not be demonstrated (73, 74).

### Tight clustering of extracellular BP180 epitopes recognized by bullous pemphigoid autoantibodies and A highly sensitive enzyme-linked immunosorbent assay for the detection of circulating anti-BP180 autoantibodies in patients with bullous pemphigoid and Serum levels of autoantibodies to BP180 correlate with disease activity in patients with bullous pemphigoid

During his postdoc period in the lab of Dr. G. Giudice, Milwaukee, WI, USA, Detlef Zillikens revealed that approximately 90% of patients with bullous pemphigoid have circulating autoantibodies against the 76-amino-acid-long 16th non-collagenous domain just outside the transmembrane domain of BP180 (35). Based on its recombinant NC16A domain, he developed a sensitive and specific ELISA system for serum autoantibodies against NC16A in patients with bullous pemphigoid and pemphigoid gestationis (34). Applying this ELISA, he was then the first to show that serum anti-BP180 NC16A IgG correlates with disease activity in bullous pemphigoid (43). These data were later the basis for the development of a widely available commercial BP180 NC16A ELISA (13).

### Autoantibodies to BP180 associated with bullous pemphigoid release interleukin-6 and interleukin-8 from cultured human keratinocytes

For the first time, an FcγR-independent effect of anti-BP180 antibodies was demonstrated when a signal-inducing event leading to the secretion of IL-6 and IL-8 was observed upon the binding of BP180-specific IgG to cultured human primary keratinocytes (38). The release of IL-8 could subsequently be shown to be significantly inhibited by dapsone but not tetracycline (75).

### IgG4 and IgE are the major immunoglobulins targeting the NC16A domain of BP180 in bullous pemphigoid: Serum levels of these immunoglobulins reflect disease activity and Correlation of serum levels of IgE autoantibodies against BP180 with bullous pemphigoid disease activity

The initial publication was the first to describe IgE reactivity against BP180 NC16A in patients with bullous pemphigoid (42). The article by van Beek et al. confirmed the previous finding that serum levels of IgE against BP180 NC16A parallel the extent of the disease interindividually, i.e., in an individual bullous pemphigoid patient during the course of the disease (76). Unlike serum anti-BP180 NC16A IgG, IgE against this domain does not correlate with disease activity per se. In addition, while anti-BP180 NC16A IgG is associated with the clinical phenotype, i.e., with the classical picture represented by blisters and erosions, IgE against this domain is unrelated to both the classical and urticarial/erythematous phenotypes.

### Autoantibodies to bullous pemphigoid antigen 180 induce dermal-epidermal separation in cryosections of human skin and Granulocyte-derived elastase and gelatinase B are required for dermal-epidermal separation induced by autoantibodies from patients with epidermolysis bullosa acquisita and bullous pemphigoid

In this *ex vivo* model, autoantibodies against the NC16A domain of BP180 from patients with bullous pemphigoid bind in cryosections of human skin and activate normal human neutrophils, which results in dermal–epidermal separation (51). Subsequently, it was shown that the activation of neutrophils leads to the release of specific proteolytic enzymes, which are pathogenically involved in the autoantibody-induced granulocyte-dependent dermal–epidermal separation (77).

### Protein A immunoadsorption: A novel and effective adjuvant treatment of severe pemphigus and Rituximab for treatment-refractory pemphigus and pemphigoid: A case series of 17 patients and Treatment of severe pemphigus with a combination of immunoadsorption, rituximab, pulsed dexamethasone and azathioprine/mycophenolate mofetil: A pilot study of 23 patients

Detlef Zillikens and his team, initially in Würzburg and then in Lübeck, were among the first to systematically apply immunoadsorption and rituximab as novel treatment options for severe AIBDs (78). The protocol combines the short-term effects of immunoadsorption and i.v. dexamethasone pulses with the long-term effects of rituximab, in addition to azathioprine/mycophenolate mofetil, and became the new standard therapy for severe and/or refractory pemphigus patients in Lübeck and offered an improved side-effect profile compared with the former standard therapy of high-dose oral corticosteroids plus azathioprine/mycophenolate mofetil (65, 79, 80).

### Induction of dermal-epidermal separation in mice by passive transfer of antibodies specific to type VII collagen

As another milestone in pemphigoid research, Detlef Zillikens provided *in vivo* proof of the autoimmune nature of epidermolysis bullosa acquisita (EBA). The story behind the paper illustrates that serendipity is sometimes a significant contributor to success: the initial aim of the project was the development of a mouse model for bullous pemphigoid. However, the murine BP180 NC15A domain was challenging to produce, while in a separate project, the production of proteins of the NC1 domain of murine collagen type VII (COL7) was successful. Hence, IgG from rabbits immunized with recombinant fragments of COL7, when injected into wild-type mice, induced clinical, histological, and immunological phenotypes duplicating human EBA (52). In parallel, similar observations were independently made by David Woodley at almost the same time (81). Ever since its initial description, this antibody transfer-induced (“passive”) EBA mouse model, or variations thereof, has been extensively used by multiple laboratories for basic and translational research in EBA and pemphigoid diseases in general (82–89).

### Anti-inflammatory activity of IgG1 mediated by Fc galactosylation and association of FcγRIIB and dectin-1

The requirement of C5 for the induction of experimental EBA was already described in the initial description of the antibody transfer-induced EBA mouse model (see above) (52). In cooperative work within the Excellence Cluster *Inflammation at Interfaces*, the groups of Detlef Zillikens and Jörg Köhl identified a novel pathway blocking C5aR1-driven inflammation. In this work, they demonstrated that i) C5aR1-deficient mice, like C5-deficient mice, are almost completely protected from clinical disease manifestations in antibody transfer-induced EBA; ii) engagement of the FcγRIIB and dectin-1 by highly galactosylated immune complexes blocks signaling downstream of the C5aR1; and iii) clinical disease manifestations can be ameliorated by treatment of mice with highly galactosylated immune complexes in the antibody transfer-induced EBA mouse model (90).

### Serological diagnosis of autoimmune bullous skin diseases: prospective comparison of the BIOCHIP mosaic-based indirect immunofluorescence technique with the conventional multi-step single test strategy

Here, a multivariant indirect immunofluorescence test based on the Biochip^®^ technology employed six miniature substrates, i.e., monkey esophagus, primate salt-split skin, recombinant BP180 NC16A, and HEK293 cells recombinantly expressing desmoglein 1, desmoglein 3, and BP230 on their cell surface in a single incubation field of a normal laboratory slide. This approach allowed the serological diagnosis of more than 90% of sera from patients with AIBDs (19). Subsequently, these findings could be corroborated using an extended Biochip^®^ mosaic (24). Meanwhile, Biochips^®^ with cells expressing type VII collagen and laminin 332 are widely available (20, 22).

### Population-specific association between a polymorphic variant in ST18, encoding a pro-apoptotic molecule, and pemphigus vulgaris

In this study in cooperation with Prof. Eli Sprecher, Tel Aviv, the first non-HLA-related gene, ST18, associated with pemphigus was described in Israeli and Egyptian patients but not in patients of German ancestry (91).

### Genome-wide mapping of gene-microbiota interactions in susceptibility to autoimmune skin blistering

In this collaborative study with John Baines, from the Max Planck Institute for Evolutionary Biology, Plön, Germany, the role of skin microbiota in the pathogenesis of AIBDs was first systemically studied (92). The study also provided evidence for host gene–microbiota interactions contributing to disease risk in a mouse model of EBA. The study was followed by several others (93, 94), eventually identifying multiple species involved in AIBDs and the metabolites mediating their effects.

### The leukotriene B_4_ and its receptor BLT1 act as critical drivers of neutrophil recruitment in murine bullous pemphigoid-like epidermolysis bullosa acquisita

In mouse models of bullous pemphigoid-like EBA and of bullous pemphigoid mice deficient in 5-lipoxygenase, a key enzyme in LTB4 biosynthesis, or BLT1, was resistant to anti-COL7- and anti-COL17 IgG-mediated neutrophil recruitment and, consequently, skin inflammation (95). A pronounced parallel increase in LTB4 and neutrophils in the skin was seen, while eosinophils appeared to be dispensable for blister formation in the bullous pemphigoid-like EBA mouse model. These data highlighted LTB4/BLT1 as critical drivers of antibody-induced tissue destruction in experimental bullous pemphigoid-like EBA and bullous pemphigoid.

### Experimental laminin 332 mucous membrane pemphigoid critically involves C5aR1 and reflects clinical and immunopathological characteristics of the human disease

This article describes the first mouse model for mucous membrane pemphigoid that reflects major clinical and immunological characteristics of the human disease (96). In this model, like in patients with mucous membrane pemphigoid, dapsone significantly reduced skin and oral lesions and ocular inflammation that leads to fibrosis (97, 98). This model will be instrumental in characterizing key inflammatory pathways and potential novel therapeutic agents for this devastating disease.

### A sensitive and specific assay for the serological diagnosis of anti-laminin 332 mucous membrane pemphigoid

Based on the Biochip^®^ technology, the first widely available detection system for serum autoantibodies against laminin 332 was developed and validated in a large multicenter study (22). This study corroborated the previous finding that anti-laminin 332 IgG in mucous membrane pemphigoid is associated with a malignancy in about a quarter of patients (99) and led to the recommendation of testing for anti-laminin IgG in all patients with mucous membrane pemphigoid in national and international guidelines (100, 101). Subsequently, N.v.B. et al. calculated a 6.8-fold higher risk of patients with anti-laminin 332 mucous membrane pemphigoid to develop malignancies than the age- and sex-matched general population (102). This association and the autoimmunity against laminin 332 are further detailed in another article on this research topic (103).

## International relations

Detlef Zillikens spent several years in the laboratory of Luis Diaz at the Medical College of Wisconsin in Milwaukee, USA, where he met European postdoctoral fellows such as José Mascaro Jr. or F.C. He worked with scientists, namely, George Giudice, who cloned BP180, and Zhu Liu. At that time, he mapped epitopes on BP180 of bullous pemphigoid, pemphigoid gestationis, lichen planus pemphigoides, and linear IgA disease. He kept life-long connections with these colleagues from this period abroad.

**Figure 5:**
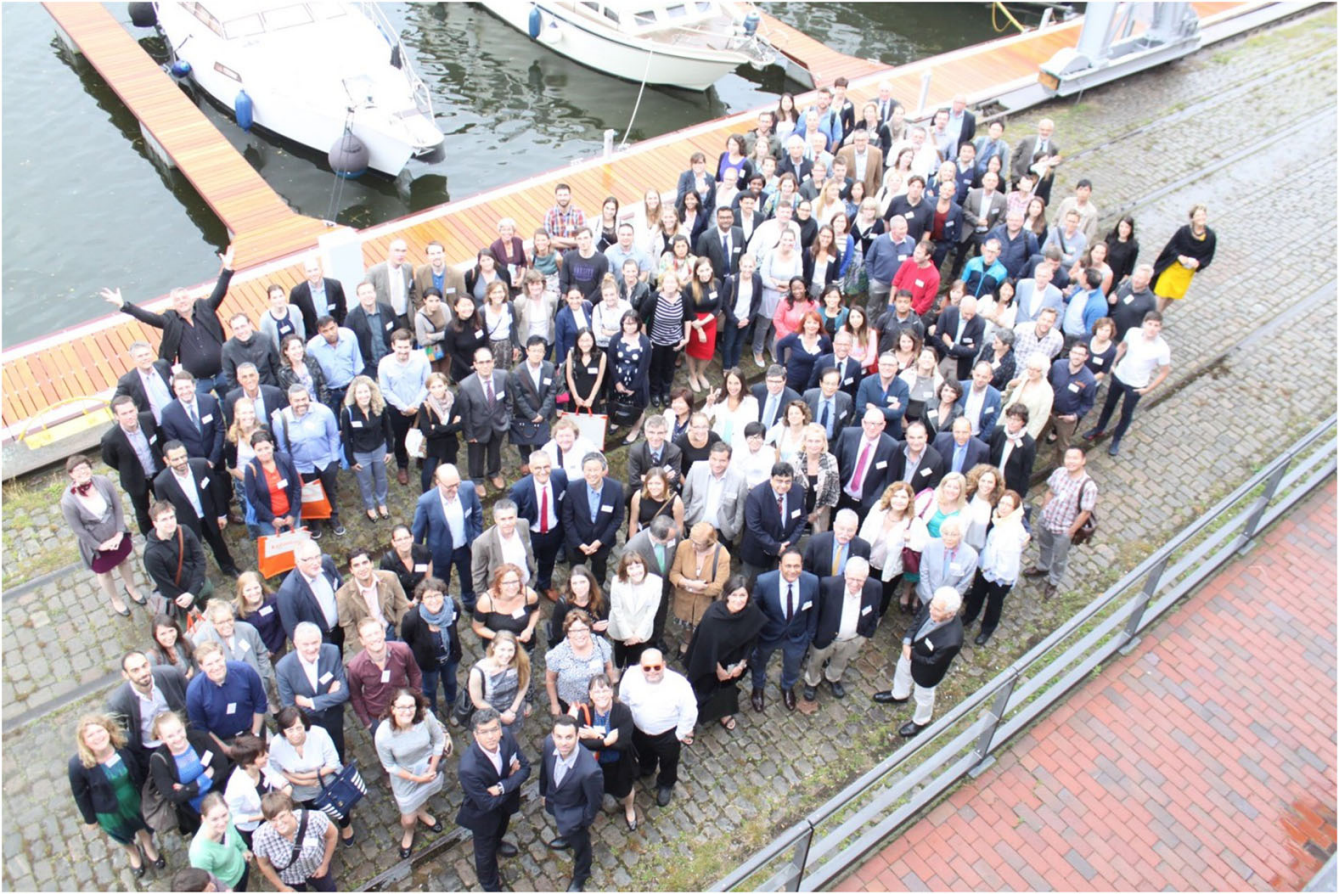
The International Pemphigus & Pemphigoid Foundation (IPPF) Meeting in Lübeck, Germany, 2017.

Detlef Zillikens organized and hosted two large international meetings on AIBDs in Lübeck, the first in 2013 before the International Investigative Dermatology (IID) meeting in Edinburgh. A chartered aircraft, known as the “Detlef Zillikens Express”, flew everyone from the local airport in Lubeck to Edinburgh. With Detlef Zillikens, nothing was impossible. The second took place in 2017 with the help of the American patients’ association International Pemphigus & Pemphigoid Foundation (IPPF) (**Figure 5**). These reunions occurred in a friendly atmosphere, permitted many exchanges of ideas and contact between people, and are still well-remembered in the entire community.

Detlef Zillikens has always welcomed students from abroad so that they can learn or do research on AIBDs in his laboratory. He also regularly received visiting professors in his department for teaching and scientific fostering including DFM, F.C., and T.H.

From an early age, Detlef Zillikens had a passion for exploring the world and meeting people. He spent several months during his medical school at the University of Illinois in Urbana, USA, where he perfected his Midwestern American accent. He returned to the Midwest, to the University of Wisconsin, where he was mentored by another world traveler physician-scientist, Dr. Luis Diaz, originally from Peru. Through this thread, Detlef Zillikens became part of a far-reaching international team of mentees of Luis Diaz who were passionate about AIBDs. Once he had established his chairmanship in Lübeck, he was then able to draw in numerous German experts and also many of the best physician scientists from abroad with grant funding to support them including Norito Ishii (University of Kurume, Japan) Hiroaki Iwata (University of Gifu, Japan) and Kentaro Izumi (University of Sapporo, Japan).

**Figure 6:**
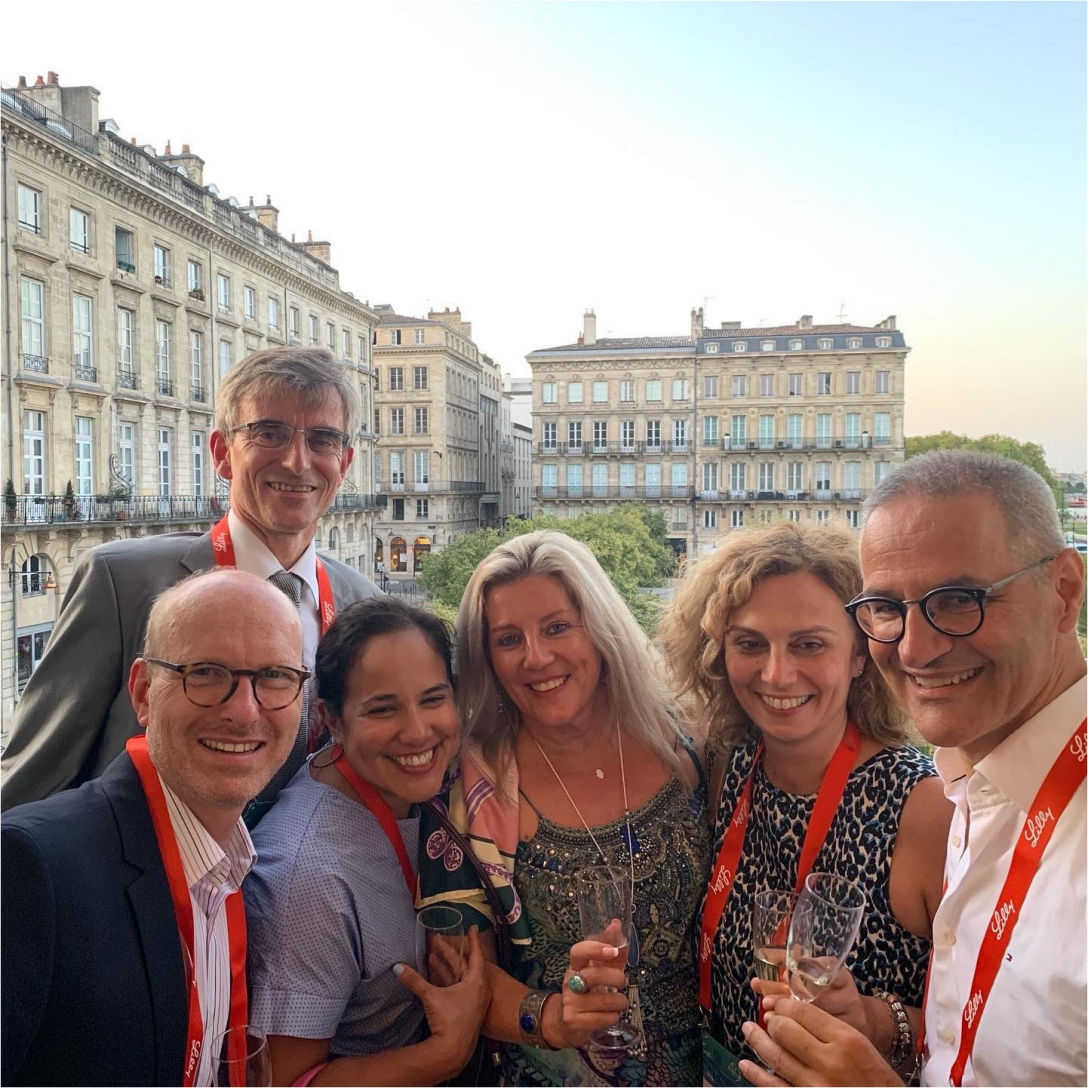
Detlef Zillikens (right) with Enno Schmidt, Frédéric Caux, Dolca Thomas, Dedee Murrell, and Katerina Patsatsi (from left to right) at the annual meeting of the European Society of Dermatological Research, Bordeaux, France, 2017.

Detlef Zillikens has always enjoyed networking and missed a few annual meetings of the German Society of Dermatological Research (ADF) and the European Society of Dermatological Research (**Figure 6**). He managed to find the time to lecture in far-flung corners of the world, including India and Iran, where he was constantly swamped by young dermatologists who were hoping to work with him.

Nevertheless, somehow, he also managed to fit in amazing trips to exotic locations with his family for their passion for swimming with tropical fish.

## Personal notes and anecdotes

Detlef Zillikens has always been an extremely hard-working person who dedicated large parts of his spare time to his clinic and research on AIBDs. This may be exemplified when, at a time when he corrected manuscripts by hand on the printed version, he occasionally handed the reviewed manuscript back, which appeared slightly wrinkled and rippled. With an exclusive smile he added, “Oh, I am so sorry, I went to the sauna”. It was rare, even when traveling with Detlef Zillikens, that he completely relaxed and took more than a few minutes to talk about private matters. E.S. always remembered one of these rare occasions when sitting in the evening light in view of the Alhambra castle during the Autoimmune Symposium 2012 in Granada, Spain. Strangely enough, Detlef Zillikens mentioned this evening during our last encounter in the rehabilitation clinic in July 2022.

M.G. first met Detlef Zillikens in 1993 when he started his dermatology residency at the Department of Dermatology, University of Würzburg. Detlef Zillikens was his first attending: he introduced M.G. into general dermatology and stringently supervised his clinical activities and rigorously corrected his medical reports. M.G. remembers the daily medical rounds (“Mittagsvisiten”) where Detlef, together with Prof. Eva-Bettina Bröcker (at that time chair of the department), Henning Hamm, Boris Bastian (now San Francisco, CA, USA), E.S. and C.R. (now both in Lübeck), and others contributed to an extremely dynamic working atmosphere that fostered intensive clinical and scientific discourse, in which M.G., among many others, was fortunate enough to complete his training. Detlef Zillikens inspired M.G. for AIBDs, and when Detlef Zillikens went to the USA, M.G. was allowed to represent him in this field in Würzburg. Returning to Würzburg, Detlef Zillikens expanded the clinic’s autoimmunology diagnostic laboratory, introduced new therapeutic approaches for AIBDs such as immunoadsorption, and was one of the first to use rituximab for AIBDs. Detlef Zillikens and M.G. formed a clinical tandem, which gave both of us considerable freedom for our research interests. This concept was implemented by Prof. Eva-B. Bröcker, which she also introduced for her oncology senior physicians, preceded today’s demand for academic support of specialists and senior physicians (now called advanced clinician scientists) by two decades and may have inspired Detlef Zillikens to introduce something comparable in Lübeck. When M.G. became chair of the Dermatology Department in Würzburg, both not only continued their long-standing scientific cooperation with Detlef but also regularly exchanged on the promotion of young scientists and discussed information on clinic matters and key operational figures of their departments. With the passing of Detlef Zillikens, M.G. has lost a passionate teacher and close friend to whom he owes a lot.

Detlef Zillikens has been the exemplary mentor of M.K. for clinical and experimental dermatology at the University of Lübeck for over a decade. With Detlef’s most dedicated help, M.K. was able to achieve his academic goals starting from board certification in both dermatology and dermatopathology up to his current professorship at the University of Southern California, Los Angeles, CA, USA. The knowledge that M.K. gained from him concerning the diagnosis and treatment of AIBD patients has been most valuable, and M.K. feels honored to share it now with his new dermatology team including Prof. David Woodley (another well-known clinician/researcher in the field of AIBDs and close colleague of Detlef Zillikens). M.K. remembers well the moment when he started his career at the University of Lübeck. He initially asked Prof. Stefania Jabłońska, Warsaw, Poland, a pioneer in immunodermatology, for advice regarding a high-ranking academic place with a focus on immunobullous diseases. Her answer was, “Prof. Zillikens’ clinic, but you would need to work hard”. This hard work has been more than rewarding to M.K. M.K. will always mourn the loss of Detlef Zillikens and remember him as a monumental figure in his life.

Detlef Zillikens and T.H. were close friends for a long time starting in 1994 and intensified further following our landmark paper about the first case of anti-200 pemphigoid (see above) (71). Detlef Zillikens and T.H. have published 53 manuscripts together in total. T.H. still well remembers when visiting Detlef Zillikens in Würzburg, the dinner of delicious asparagus in the restaurant overlooking the city of Würzburg. Detlef Zillikens and his wife Monika also treated T.H. in their home with very nice German food. More recently, in 2017, T.H. had a 1-month sabbatical at Lübeck owing to Detlef Zillikens’ help. T.H. prays that Detlef’s soul rests in peace.

In conclusion, we have lost not only a giant in dermatology but also a wonderful and kind mentor and friend, Detlef Zillikens, to whom this Research Topic of *Frontiers in Immunology* is dedicated.

## Data availability statement

The original contributions presented in the study are included in the article/supplementary material. Further inquiries can be directed to the corresponding author.

## Ethics statement

Written informed consent was obtained from the individual(s) for the publication of any potentially identifiable images or data included in this article.

## Author contributions

JEH: Conceptualization, Writing – review & editing. CDS: Writing – review & editing. NvB: Writing – review & editing. HB: Writing – review & editing. FC: Writing – review & editing. MG: Writing – review & editing. CMH: Writing – review & editing. KH: Writing – review & editing. TH: Writing – review & editing. SI: Writing – review & editing. MK: Writing – review & editing. DFM: Conceptualization, Writing – original draft, Writing – review & editing. AR: Writing – review & editing. CR: Writing – review & editing. NS: Writing – review & editing. IS: Writing – review & editing. CS: Writing – review & editing. PT: Writing – review & editing. DT: Writing – review & editing. RJL: Writing – review & editing. ES: Conceptualization, Supervision, Writing – original draft, Writing – review & editing.
